# Potential of *Spirulina platensis* as a feed supplement for poultry to enhance growth performance and immune modulation

**DOI:** 10.3389/fimmu.2023.1072787

**Published:** 2023-01-31

**Authors:** Nahed A. El-Shall, Shouqun Jiang, Mayada R. Farag, Mahmoud Azzam, Abdulaziz A. Al-Abdullatif, Rashed Alhotan, Kuldeep Dhama, Faiz-ul Hassan, Mahmoud Alagawany

**Affiliations:** ^1^ Department Poultry and Fish Diseases, Faculty of Veterinary Medicine, Alexandria University, Edfina, El-Beheira, Egypt; ^2^ Institute of Animal Science, Guangdong Academy of Agricultural Sciences, State Key Laboratory of Livestock and Poultry Breeding, Key Laboratory of Animal Nutrition and Feed Science in South China, Ministry of Agriculture and Rural Affairs, Guangdong Provincial Key Laboratory of Animal Breeding and Nutrition, Guangzhou, Guangdong, China; ^3^ Forensic Medicine and Toxicology Department, Faculty of Veterinary Medicine, Zagazig University, Zagazig, Egypt; ^4^ Department of Animal Production College of Food & Agriculture Sciences, King Saud University, Riyadh, Saudi Arabia; ^5^ Poultry Production Department, Agriculture Faculty, Mansoura University, Mansoura, Egypt; ^6^ Division of Pathology, Indian Council of Agricultural Recearch-Indian Veterinary Research Institute, Bareilly, Uttar Pradesh, India; ^7^ Institute of animal and Dairy Sciences, University of Agriculture, Faisalabad, Pakistan; ^8^ Poultry Department, Agriculture Faculty, Zagazig University, Zagazig, Egypt

**Keywords:** *Spirulina platensis*, poultry, production, immunity, antimicrobials, antiviral, anticoccidial, nutrition

## Abstract

Increase in drug resistance as well as ineffective immunization efforts against various pathogens (viruses, bacteria and fungi) pose a significant threat to the poultry industry. Spirulina is one of the most widely used natural ingredients which is becoming popular as a nutritional supplement in humans, animals, poultry and aquaculture. It contains protein, vitamins, minerals, fatty acids, pigments, and essential amino acids. Moreover, it also has considerable quantities of unique natural antioxidants including polyphenols, carotenoids, and phycocyanin. Dietary supplementation of Spirulina can beneficially affect gut microbial population, serum biochemical parameters, and growth performance of chicken. Additionally, it contains polyphenolic contents having antibacterial effects. Spirulina extracts might inhibit bacterial motility, invasion, biofilm formation, and quorum sensing in addition to acting directly on the bacterium by weakening and making the bacterial cell walls more porous, subsequently resulting in cytoplasmic content leakage. Additionally, Spirulina has shown antiviral activities against certain common human or animal viruses and this capability can be considered to exhibit potential benefits against avian viruses also. Spirulan, a calcium-rich internal polysaccharide of Spirulina, is potentially responsible for its antiviral effect through inhibiting the entry of several viruses into the host cells, boosting the production of nitric oxide in macrophages, and stimulating the generation of cytokines. Comparatively a greater emphasis has been given to the immune modulatory effects of Spirulina as a feed additive in chicken which might boost disease resistance and improve survival and growth rates, particularly under stress conditions. This manuscript reviews biological activities and immune-stimulating properties of Spirulina and its potential use as a dietary supplement in poultry to enhance growth, gut health and disease resistance.

## Introduction

Numerous diseases continue to occur in the poultry industry despite the widespread use of vaccines and medications, resulting in financial losses. For instance, avian viral infections such as avian influenza (AI), infectious bronchitis (IB), infectious bursal disease (IBD), and Newcastle disease (ND) lead to significant economic losses, particularly in broilers, due to respiratory distress, increased mortality, reduced growth, and immunological suppression (1). The same is true for coccidiosis and bacterial infections like Salmonella, E. coli, etc. Until the emergence of resistant strains, chemical antimicrobials and antiprotozoals successfully inhibited and suppressed bacteria and protozoa. So there is a persistent need to use natural substances to address this issue. Algae, for instance, is a source of vital biological useful components, making the use of natural habitats as a source of these chemicals a viable strategy for creating innovative cuisines (2, 3). One of the greatest sources for organic nutrients among edible algae is the microscopic blue-green alga Spirulina (Arthrospira), which is used as a nutritional supplement for both human and animal feed globally (4). *Spirulina platensis* is a filamentous commercial cyanobacterium that is utilized as dietary and feed supplement in humans, aquaculture, livestock and poultry industry. Spirulina can grow in both saline and fresh water, and it is semi- and mass-cultivated in several countries. Dried spirulina is a rich nutritional source, with a high protein content (260-770 g/kg) representing 70% dry weight and a large fat content (10-140 g/kg) (5–7). Additionally, it has been observed that these microalgae have a high nutrient digestibility that was superior or equivalent to that of other vegetable diets and feeds (6, 8). Therefore, Spirulina has the potential to partially replace the traditional protein sources, particularly soybean meal (9). Oleic acid, linoleic acid, gamma-linolenic acid, docosahexaenoic acid (DHA), sulfolipids, and glycolipids are among the many polyunsaturated fatty acids found in spirulina, in addition to Omega-3 and -6 polyunsaturated fatty acids that are abundant in spirulina (25% and 60% of the total fatty acids) (5, 10). Spirulina also contains pigments, such as carotenoids (4000 mg/kg), which include β-carotene and zeaxanthin (10, 11), and chlorophyll pigments (12–14). Phycobiliproteins (15), vitamins (16), and macro- and micromineral components like calcium, iron, magnesium, manganese, potassium, zinc, and selenium are also found in spirulina (10, 17). Moreover, polysaccharides, pro-vitamin A, vitamin E, vitamin K and various B vitamins (10) as well as antioxidants are also important constituents of spirulina (18).

**Figure 1:**
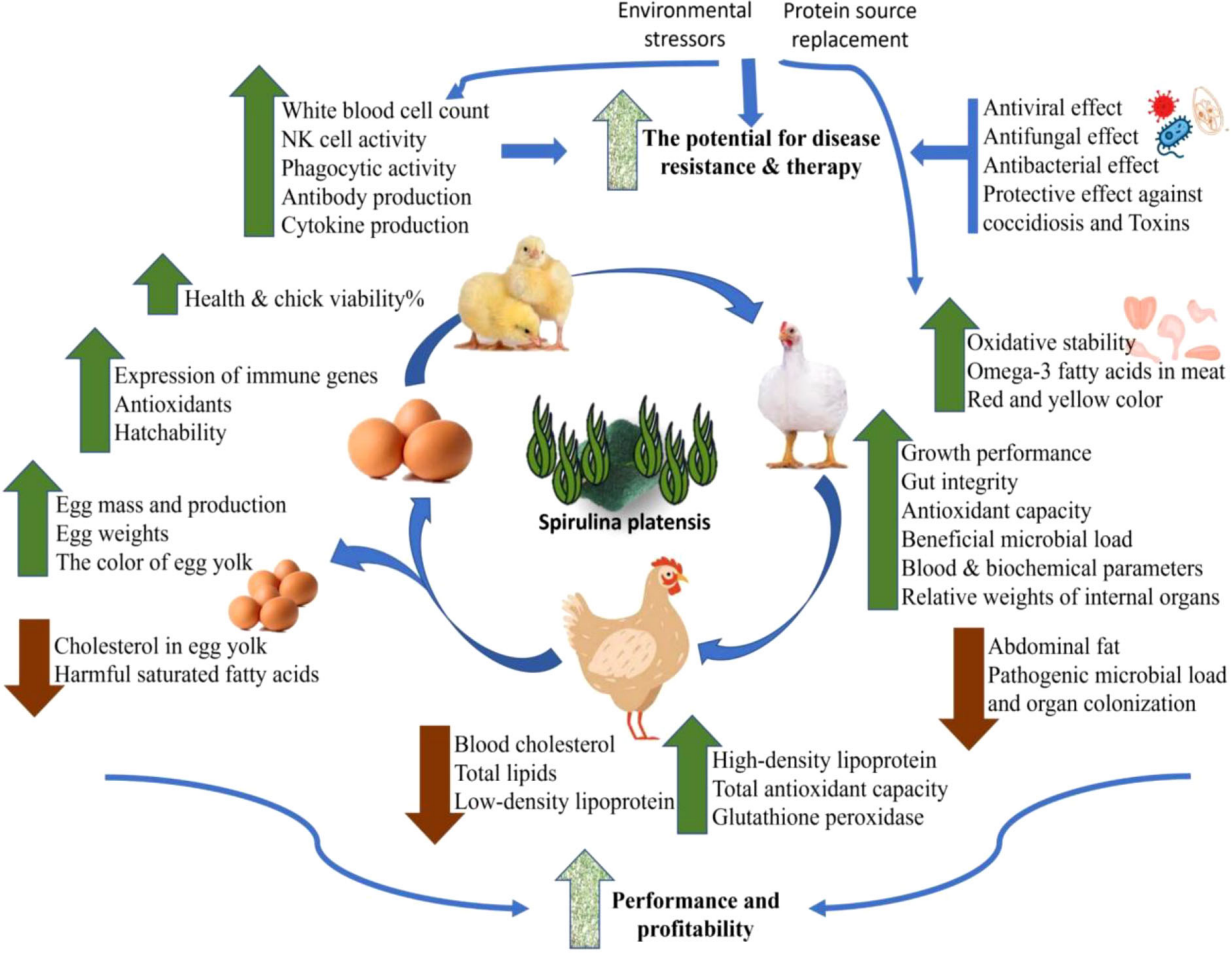
Overall applications and effects of Spirulina on poultry performance and health.

Spirulina is used as a dietary additive in a wide variety of food products due to its exceptional and impressive nutrient composition. This helps to improve the nutritional qualities of the products, as well as their potential to improve reproductive and productive performance, general health, and the symptoms of various animal diseases like arthritis, diabetes, anaemia, hypertension, and cardiovascular disorders. Spirulina are strong contenders as an alternative to antibiotics in chicken feed. These substances exhibited potential medicinal properties like antimicrobial, antioxidant, anti-cancer, anti-inflammatory, immune-enhancing, and colourants (18–20) in addition to their metalloprotective, radioprotective, and hypocholesterolemic effects (4, 10). Additionally, Spirulina (SPA) with antiviral properties has shown to strengthen the immune system, and its rich nutritional profile promoted growth performance by improving the intestinal villi length and number of the epithelial cells particlulary goblet cells (21). For a sustainable and feasible future of food security, spirulina is becoming a more affordable method of increasing poultry output (9). Here, therapeutic and immune-stimulating benefits of Spirulina were reviewed from another angle in case they could actually be used as nutritional supplement with antibiotic or vaccine to fight off various diseases of chicken (**Figure 1**).

## Effects of *Spirulina platensis* on productive performance

Numerous studies have supported spirulina’s ability to promote growth. From the embryonic stage until the egg is laid, spirulina can be supplemented in poultry diets. *Spirulina platensis* in ovo injection enhanced the expression of genes associated to immunity, antioxidants, and hatchability in quail chicks (22). Ibrahim et al. (23) reported that spirulina in drinking water for 4 weeks at levels of 0.5, 1 and 2 g/Liter significantly increased the average body weight gain, health, with the highest chick viability percent, best significant feed conversion ratio (FCR), feed efficiency, European Production Efficiency values, increase in the relative weights of carcass and internal organs with the significantly lowesr abdominal fat. Spirulina supplemented (1 or 2 g/kg diet) in diets containing vegetable and animal protein in Japanese quails improved the growth performance without affecting meat quality or gut flora in quails fed with vegetable protein source but no effect was observed in animal protein based diet (24).

K-strain White Cornell Leghorns and broiler chicks grown to ages of seven and three weeks, respectively, on diets containing varied concentrations (10, 100, 1,000, and 10,000 ppm) of *Spirulina platensis* exhibited higher body weights than control birds (25). With 0.7 and 0.9 g of *Spirulina platensis* per kg of feed, Cobb broiler chickens’ growth performance, blood parameters, biochemical changes in serum, and microbial load could all be improved (26).

Dietary supplementation of Spirulina at 10g/kg of diet showed significantly higher body weight gain and, consequently, linear improvements in FCR was observed in Cobb broilers during 35 days experimental period. Additionally, dietary Spirulina levels resulted in a rise in intestine Lactobacillus sp. while decreasing Escherichia coli populations (27).

Hajati and Zaghari (28) advocate utilizing Spirulina at dosages of 5 and 3 g/kg food during the Japanese quail’s growth and laying periods, respectively, using a range of varied doses. During the first 35 days of life, a diet of 5 g/kg induced a significant increase in body weight gain, breast percentage, and European production efficiency factor. When added to the diet of layers, Spirulina at a level of 3 g/kg considerably reduced the amount of cholesterol per gram of yolk while also improving the color of the egg yolks.

However, daily feed intake, FCR, the percentage of broken eggs, eggshell thickness, albumen height, Haugh unit, and egg weight were unaffected by dietary Spirulina up to 0.9%. When compared to non-supplemented birds, 0.6% algae significantly increased egg mass and production as well as egg yolk colour in laying chickens between 26 and 37 weeks of age (29).

For improved reproductive and productive performance, Mobarez et al. (30) suggested adding Spirulina to Golden Montazah layer diets. When given the basic diet with 2 or 3 g Spirulina/kg diet during the laying period from 29 to 40 weeks of age, hens and cocks had significantly improved FCR compared to the control group. Additionally, chickens fed a diet containing 3 g of Spirulina/kg had the highest levels of high-density lipoprotein (HDL), total antioxidant capacity (TAOC), glutathione peroxidase (GPx), egg quantity, egg weight, and hatchability percentage. Blood cholesterol, total lipids, and low-density lipoprotein (LDL) were all significantly reduced with Spirulina administration at both dosages.

Microalgae in quail diets (5%, 10%, and 15%) increased egg quality and provided benefits to consumer health by functioning as an immune-stimulant and antioxidant (31). However, they had no effect on egg production. This was because it boosted amounts of monounsaturated fatty acids, which are good for consumer health, and decreased levels of harmful saturated fatty acids. Additionally, egg yolk antioxidant levels increased, which reduced lipid peroxidation. On the other hand, 32 reported a lower performance of broiler chickens supplemented with Spirulina by 15%, for 2 weeks period (21^st^ -35^th^ day old), compared to non-supplemented birds. They attributed this negative effect to the high digesta viscosity induced by the gelation of indigestible proteins of Spirulina. Even addition of exogenous enzymes like lysozyme or Rovabio Excel AP resulted in the same worse findings, although lysozyme succeeded in breaking Spirulina’ cell wall. Pestana and hos co-authors suggested that this microalga’s proteins may be more easily digested and prevented from harmfully gelling if lysozyme and an exogenous specialised peptidase were combined.

## Effects of *Spirulina platensis* on the immune system

Supplementing with spirulina boosts many immune processes. Spirulina has shown a specific action on monocytes and natural killer (NK) cells, which are essential components of the innate immune system. Administration of Spirulina exhibited to enhance macrophage phagocytic response and activity of NK cells in chicken and humans (25, 33, 34). The phagocytic activity of macrophages isolated from cats was also found to be increased in response to antigen exposure in the presence of Spirulina (25). A polysaccharide extract of *Spirulina platensis* has hsown to increase white blood cells in a haematopoietic system damaged by irradiation (35). Oral administration of *Spirulina platensis* in healthy male volunteers increased IFN-γ production and phagocytic activity of isolated NK cells stimulated with IL-12/18. Beside this, Spirulina also enhanced Toll like receptor (TLR)-2 and 4 mediated production of IL-12 from peripheral blood mononuclear cells, thus indicating Spirulina first activates monocytes and macrophages to produce cytokines that stimulate NK cells (34). An action through TLR-2 or -4, leading to NF-κB activation, has been suggested in studies in human monocytes (36, 37).

Spirulina’s immune-modulatory action on mice through increased IL-1 antibody production was observed in 1994 for the first time (38). In this regard, Kaoud (39) found that chicken groups fed diets containing spirulina had higher relative and absolute thymus and bursa weights than the control group. Similarly, in comparison to the untreated control, the addition of *S. platensis* at levels of 0.7 and 0.9 g/kg broiler diet resulted in a considerable rise in the weights of the bursa, thymus, and spleen (26). However, broilers and K-strain chicks given Spirulina (0, 10, 100, 1,000, and 10,000 ppm) did not change in bursal or splenic weight, but the K-strain chicks had significant larger thymuses than the controls (25).

A considerable rise in white blood cell count and increased macrophage phagocytic activity in broilers treated with *S. platensis* algae suggested that the immune system of the animals was strengthened (40).

According to Al-Batshan et al. (33) feeding *Spirulina platensis* increases macrophage phagocytic activity in terms of the average number of sheep red blood cells (SRBC) per phagocytic macrophage (range = 2.2 to 3.6 versus 1.8 to 2.5 in the basal group) and the overall phagocytic percentage (range = 28 to 39% versus 24 to 25% in the basal group). Over the course of the three developmental ages, Escherichia coli lipopolysaccharides-induced nitrite levels in macrophages (increased nitric oxide synthase activity) ranged from 60 to 278 microM in the basal diet group, but they were significantly higher in all Spirulina dietary groups (0.5% group range = 198 to 457 microM; 1.0% group range = 161 to 359 microM; and 2.0% group range = 204 to 420 microM).

Spirulina supplementation at 10000 ppm of diet also doubled the activity of NK cells and showed a greater PHA-P-mediated lymphoproliferative response compared to controls. All Spirulina groups (10, 100, 1000, 10000 ppm) demonstrated greater macrophages phagocytic capacity than the 0 ppm group in K-strain and broilers (25).

When compared to 1 g Spirulina/kg, laying Japanese quails given 3 or 5 g Spirulina/kg had significantly enhanced cutaneous basophil hypersensitivity after 12 or 24 hours of phytohemagglutinin injection (28). A substantially less heterophil and more lymphocytes than the control treatment were obtained by 1, 1.5, and 2 g spirulina/kg fed to broilers for 42 days (21). It has been demonstrated to increase the potential for disease resistance in chicken by activating their mononuclear phagocytic system (41). Improvement in cellular immunity observed in response to dietary supplementation of Sprirulina might be attributed to higher Zn concentration in spirulina like this (42, 43).

In Cornell K-strain White Leghorns and broiler chicks fed to 7 and 3 weeks of age, respectively, with meals containing varying amounts of *Spirulina platensis* (0, 10, 100, 1,000, and 10,000 ppm), anti-sheep red blood cell antibodies were not different throughout the initial reaction. However, all Spirulina-dietary groups with K-strain chicks exhibited greater total anti-SRBC titers during the secondary response, with the 10,000-ppm group having the highest (6.8 Log2) compared to the 0 ppm (5.5 Log2) group (25). In laying Japanese quails, different concentrations of Spirulina (1, 3 or 5 g/kg food) resulted in significantly greater levels of total antibody against SRBC and IgG titers (28).

Khan et al. (21) showed that the use of spirulina considerably enhanced growth performance, gut integrity, and immunity in broiler production while also providing better economics and supplementing with spirulina significantly affected the antibody titer against the ND vaccination.

In growing Japanese quail chicks, adding Spirulina at concentrations of 0.5, 1 and 2 g/Liter significantly raised the amount of serum antibodies against the Newcastle virus (NDV) and the plasma total protein profile (23).

Additionally, Golden Montazah laying hens and cocks supplemented with 3 g Spirulina/kg diet during the laying period recorded significantly higher antibody titers against NDV, Avian Influenza (AI), antibody against SRBC, and Interferon proteins (IFN- γ) concentration (30). Similarly Nia et al. (29) revealed that Spirulina by levels 0.3%, 0.6% or 0.9% in Lohmann Selected Leghorn (LSL) laying hens between the ages of 26 and 37 weeks had a substantial impact on the antibody titer in birds that had received the Newcastle vaccine. However, the ratio of heterophiles to lymphocytes, humoral immunity against SRBC, cell-mediated immunity response to PHA injection, and the relative weight of the bursa and spleen were not significantly affected by this dietary Spirulina.

Additionally, the immunosuppressive effects of diclofenac sodium, which were manifested by decreased phagocytic activity, phagocytic index, and a significant decrease in the titer of antibodies formed against NDV were significantly reversed by oral supplementation of broiler chicks with Spirulina at a dose of 10 gm/kg in their diet, either on therapeutic or preventive regimes (44).

## Antioxidant effects and *Spirulina platensis’s* impact on meat quality

Because microalgae are a significant source of C-phycocyanin, an antioxidant pigment with hypolipidemic activity, birds fed Spirulina showed improved antioxidant activity, which is another benefit of Spirulina feed (45, 46). Phycocyanin, carotene, and xanthophyll phytopigments, tocopherols, linolenic acid, and phenolic compounds are some examples of naturally occurring components in Spirulina that have been demonstrated to have strong antioxidant properties and potent scavenging activities against Reactive Oxygen Species like superoxide and hydrogen peroxide radicals (4).

Abdelkhalek et al. (47) reported that spirulina dramatically stimulates the activity of antioxidant enzymes preventing lipid peroxidation, DNA damage, and free radical scavenging. The total antioxidant capacity and thyroxin (T4) content were dramatically enhanced in growing Japanese quail chicks when Spirulina levels of 0.5, 1 and 2 g/Liter were used. Plasma cholesterol, total lipids, ALP, ALT, and AST activity, however, had significantly lower levels (23).

Additionally, in ovo injections of Arthrospira (Spirulina) platensis at doses of 0.75–3.5 mg/egg in Japanese quails and 25–35 mg/egg in broiler breeders enhanced chick hatchability and IFN-gamma expression. In quails, 2.5 or 3.5 mg Spirulina dramatically reduced expression of HSP70 and considerably boosted Catalase activity and GPX gene expression in hatchlings. The lowest HSP70 in chicks was induced by in ovo injection of 25 or 35 mg Spirulina in broiler breeders (22).

Moreover, spirulina inclusion in the poultry feed affects meat quality parameters like color, flavor, polyunsaturated fatty acids composition, and oxidative stability. Therefore, in terms of oxidative stability and the enhancement of omega-3 fatty acids like those of linolenic and docosahexaenoic acid by Spirulina feeding, 1.5% fed to Cobb 500 broiler chicks for 5 weeks could be potential functional ingredients to generate value-added broiler meat (breast and thigh meat) (48). Similarly, Abbas et al. (49) demonstrated that 3% and 4% of dietary Spirulina significantly increased the content of oleic acid, palmitic acid, docosahexaenoic acid, and linoleic acid in broiler carcass. Additionally, throughout the 30-day storage period, 4% Spirulina considerably decreased the value of peroxide. For the 60-day storage period, both Spirulina treatments significantly reduced the oxidation indices of total volatile nitrogen (TVN) and thiobarbituric acid (TBA), while the sensory evaluation ratings for the chest and thigh slices did not change. 50 reported an unchange of the amount of gamma-linolenic acid or omega-3 in the intramuscular fat by 75% and 50% Soy bean replacement by Spirulina in starter and grower feed, respectively, although the richness of Spirulina with poly unsaturated fatty acids. Moreover, when Spirulina-based meat samples were wrapped in highly oxygenated modified environment packaging, they showed higher rates of lipid oxidation than soybean meal-based meat samples (50). On another side, color of meat was improved by inclusion of spirulina at high doses in the feed that may be due to high level of carotenoids. Altmann et al. (51) found that when Spirulina replaces 50% of the soy protein in broiler diets, meat color could be increased to be dark reddish-yellowish flesh. Similarly, Altmann et al. (50) observed a more intensive color (higher red (a*) and yellow (b*)) for breast and thigh meat as well as an increased umami and chicken flavor were reported for broiler meat. 51 reported a positive effect of Spirulina on flavour breast filets through decreasing the score of metallic flavor (off-flovor) of breast meat, besides increasing pH value after 24 hours after death that was associated with softness and tenderness of breast filets. Feeding broiler chickens with Spirulina 15% for two, the breast and thighs exhibited higher values of yellowness (b*) compared to the control, as well as total carotenoids and saturated fatty acids were increased, but levels of n-3 polyunsaturated fatty acids and α-tocopherol were reduced (32). Neverthless, Park et al. (52) found that the quality of the breast meat of broilers fed Spirulina diets (0.25, 0.5, 0.75, or 1.0%) for 35 days was not substantially altered while seven-day drip loss was linearly reduced.

## Anti-pathogenic effects of *Spirulina platensis*

Dietary spirulina have shown good response in several infections *in vivo* in poultry (**Table 1**). A wide variety of influenza viruses, including oseltamivir-resistant strains, were blocked by the cold-water Spirulina extract from forming viral plaques. Inhibition of influenza hemagglutination was revealed to be one of the ways by which spirulina extract acts early in the course of infection to lower viral production in cells and increase survival in influenza-infected mice (58). The ethanol extract of *Spirulina platensis* had an antiviral effect *in vitro*, decreasing the infectious units of adenovirus types 7, Coxsackievirus B4, astrovirus types 1, rotavirus Wa strain, and adenovirus types 40 by respective amounts of 53.3%, 66.7%, 76.7%, 56.7%, and 50% (59).

**Table 1:** Efficacy of *Spirulina platensis* (SP) supplementation in cases of various infectious diseases in poultry.

Algae/dose	Route of administration/duration	Birds	Vaccination/Age	Infectious challenge/Age/dose/route	Main results	Reference
Avian Influenza						
*S. platensis* 0%, 10%, 20%	Drinking water/7^th^ day to 32^nd^ day	Broiler chickens	No vaccine	Avian Influenza H5N1/26 days old/0.1 ml inoculum containing 10^7^ EID_50_/nose drops	Myocardial necrosis did not differ significantly from either group. Comparing SP 10% and 20% to a control therapy without it, more leukocytes were produced. 20% SP showed no mortality, but 0% and 10% spirulina revealed 30% mortality.	(41)
New Castle disease						
*S. platensis* 0, 0.5, 1, 1.5 and 2 g/kg of ration	Feed at 7 days of age	SPF chickens	Commercial inactivated Newcastle disease (ND) vaccine at 21 days of age	NDV genotype VII/28 days post-vaccination/0.5 mL/bird containing 10^6^ EID50/intramuscular (I/M)	Compared to untreated vaccinated hens, offered adequate protection against heterologous challenge virus (90%, 100%, 100%, and 100%, respectively). Compared to the untreated group (46%), treated vaccinated hens excreted fewer viruses (55%, 65%, 76%, and 87%).	(53)
0, 1% of Microalgae (Scenedesmus obliquus, Scenedesmus quadricauda, Ankistrodesmus, Coelastrum microporum, Selenastrum, Oocystis parva, Dictyosphaerium pulchellum, Coelastrum reticulatum, Pediastrum gracillimum, Siderocells elegans, Eudorina elegans, Clamydomonas reinhardi and Micractinium pusillum (green algae group), Euglena sp. (Euglenophyta), Oscillatoria limnetica (blue-green algae group) and Nitzschia linearis (diatoms group)	Feed (1^st^ -40^th^ day of age)	Broiler chickens	▪live NDV La Sota strain by oculo-nasal route at 5^th^ day and/or ▪Inactivated NDV genotype II vaccine S/C at 18^th^ day	vvNDV genotype VIId/6-Log-10 EID50 given 0.5 ml/bird *via* I/M at 28^th^ day	The serological response, viral shedding after viral challenge, protection rate, and body weight increase of the chicken groups that received either a microalgae-free diet or one that included microalgae were comparable.	(54)
Infectious bursal disease						
Probiotic *S. platensis* 0, 1%	Feed (10^th^ -20^th^ day)	chickens	IBDV intermediate plus strain vaccine (hot strain)/17 days	–	The adverse effects of the IBD vaccine’s hot strain on blood total protein and albumin levels could be slightly mitigated by SP supplementation.	(55)
Salmonellosis						
*S. platensis* at 0, 1, and 2 g/kg diet	Feed (7^th^ day– experimental end)	quail	–	S. enteritidis/orally/21^st^ day/1ml of 1.00x10^7^)	SP dramatically increased IL-10, antioxidant and serum biochemical markers, and growth performance. It decreased the post-challenge death rate from 23.33% in the untreated group to 10% in the groups who received both doses of treatment and had significantly lower clinical symptoms. It considerably decreased organ colonization (liver, heart, spleen, caecum). The expression of the genes for serum amyloid and the pro-inflammatory IL-6, IL1 ß, and TNF-α in the cecum was considerably downregulated.	(56)
Coccidiosis						
Microalgae-derived feed ingredients at 0.175%	Feed up to 42 days old	Ross 308 broilers	–	10X Coccivac-B52 vaccine/orally/at 14^th^ day of age	Birds fed algae shed 2.3 times more oocysts than birds fed the control diet. Algal inclusion had no impact on the rate of growth and did not shield birds that had been injected with Eimeria from the modifications that Eimeria caused to their splenic T cells. However, algae significantly protected jejunal villus height as early as 7 day post infection and maintained intestinal integrity during coccidiosis.	(57)

It was reported that a calcium-rich intracellular polysaccharide called spirulan found in *Spirulina platensis* prevents multiple viruses from replicating *in vitro* by preventing the virus’ entry into the various host cells that are being utilized (60, 61), increases macrophage nitric oxide synthesis and stimulates cytokine production (62). Broiler hens infected with the H5N1 Avian Influenza virus had cardiac necrosis, although 20% of spirulina had no discernible effect on this. However, it boosted leukocytes associated with an immune function, which prevented mortality vs 30% death in the non-supplemented group (41).

Regarding Newcastle disease, most investigations such as (53, 54) found that Spirulina had an impact on viral challenge in birds that had received live, attenuated, and/or inactivated vaccinations. As a result, the effect of Spirulina supplementation was seen as an immune-stimulating effect that dramatically improved clinical protection against heterologous strains and the capacity to decrease NDV shedding. In 2019, Kumari et al. discussed an indirect benefit of spirulina on infectious bursal disease. The decreasing effect of intermediate plus vaccine (hot strain given at the age of 17 days) on serum total protein concentration was greatly reduced by spirulina supplementation at 1.0% in feed from 10 to 20 days of age.

Cyanobacteria might be considered a suitable source for the manufacturing of antimicrobial agents as purified antimicrobial compound produced by *S. platensis* were more effective against Gram positive, Gram negative, and C. albicans, a unicellular fungus. Organic and aqueous extracts of *S. platensis* were tested *in vitro* and demonstrated broad-spectrum antibacterial and antifungal action. 63 showed that the highest biological activity of them was against Escherichia coli, Pseudomonas aeruginosa, Bacillus subtilis and Aspergillus niger. Compared to ethanol or aqueous extracts, methanol extract exhibited superior antibacterial activity against all tested bacterial strains (Gram positive bacteria, Gram negative bacteria, and Candida sp.), particularly against Gram positive bacteria (Staphylococcus aureus, Streptococcus pneumoniae, Bacillus cereus, and Enterococcus faecalis). For the various strains examined, the lowest inhibitory concentration value of ethanol and methanol extract ranged between 5-100 mg/mL (64). Similarly, compared to other extracts, the methanolic extract of Spirulina showed a larger total phenolic content and more antibacterial activity (65, 66).

Kaushik and Chauhan (67) reported that the minimum inhibitory concentrations (MIC) of the methanol extract against S. aureus and E. coli were 128 g/ml and 256 g/ml, respectively while it had no effect was against Klebsiella pneumoniae. Nevertheless, Spirulina acetone extract shown strong biological activity against Klebsiella pneumoniae and modest activity against Salmonella typhi and Pseudomonas aeruginosa (68). By using an ethanol extract of *Spirulina platensis*, inhibitory zones against Enterococcus faecalis and Candida albicans were seen using the disc diffusion technique (59). The effectiveness of Spirulina’s antibacterial actions *in vivo* was assessed by Abd El-Dayem et al. (56) as adding 1 and 2 g of spirulina per kg of diet significantly increased growth performance, antioxidant levels, and the production of the anti-inflammatory cytokine (IL-10) while reducing organ colonization and gene expressions of IL-6, IL-1ß, and TNF-α as compared to positive and negative control groups.

According to 69, pathogens use the same adhesion and invasion mechanisms to invade the guts of both people and animals, and the antibacterial action of spirulina might be attributed to its ability to prevent pathogen motility, invasion, biofilm formation, and quorum sensing. In addition, Spirulina’s bioactive ingredients have shown to weaken bacterial cell walls making it more permeable, that subsequently led to leakage of cytoplasmic contents (65).

Furthermore, when broilers were given the 10X Coccivac-B52 vaccine orally while being fed microalgae-derived feed components, the intestinal integrity of the birds during coccidiosis was conserved, and the jejunal villus height was protected as early as 7 days after the challenge (57). However, the algal element changed the immune response (splenic T cells) in a way that decreased recruitment from secondary lymphoid organs (57).

Spirulina was also a powerful binder for aflatoxins in broiler breeders (70). The negative effect of 300 ppb aflatoxin on broiler chicken growth rate and lymphoid organ weights might be partially mitigated by the addition of spirulina at a level of (0.05%) although there was no positive impact on feed consumption, the serum protein concentration, liver weights, or cholesterol levels (71). Likewise, Spirulina inclusion (0.02%) in feed had positive effects on growth, ready to cook yields, bursa weight, and cellular immune response in chicks fed aflatoxin (300 ppb) although there was no effect of Spirulina on feed intake, feed conversion efficiency, leg abnormality scores, SRBC response, and weights of the liver, giblets, spleen, and abdominal fat (72). Dietary inclusion of Spirulina is also recommended to protect against other toxins as shown in **Table 1**.

## Effects of Spirulina on the managemental and nutritional shortages

A crucial component of broiler productivity, especially with the use of antibiotics being reduced, is immune system health. Natural antibiotic alternatives and nutritional factors like crude protein % are being studied to understand how they affect immunity. The positive benefits of Spirulina in reversing the management and nutrient deficits in poultry were displayed in **Table 2**. A promising development for reducing feed costs without compromising the health of the bird is the ability of natural feed additives to counteract the negative effects that low crude protein has on immunity in birds (74). Spirulina is an alternative protein-containing component that manufacturers are considering because of both its great nutritional value and its capacity to strengthen the immune system. For instance, the addition of spirulina to low protein chicken diets resulted in reduction of systemic inflammation and bacterial translocation indicating its suitability as a alternative protein source (73). Additionally, the low crude protein (LCP) diet-Spirulina supplemented reversed the effects of the LCP diet, causing the monocyte proportions and concentrations in Ross 708 old male broilers to be similar statistically to those of the control base diet (74). However, compared to 20% crude protein without Spirulina, using a diet of 17% crude protein with 10% *S. platensis* for Ross 708 broiler females exhibited no detrimental effects on the health of broilers but substantial reduction in body weight in supplemented group led to economoic losses (75).

**Table 2:** Efficacy of *Spirulina platensis* (SP) in cases of nutritional deficiencies and managemental defects in poultry.

Algae/dose	route of administration/duration	Birds	Nutritional or managemental defect	Main results	Reference
Low protein diet					
*S. platensis* at 0, 100 g/kg	Feed/14^th^ -37^th^ day of age	Ross 708 male broiler chickens	Low protein diet (LCP) (17%) compared to basal diet (21%)/14^th^ -37^th^ day of age	SP decreased hepatic bacterial translocation brought on by a LCP and systemic inflammation based on the percentage of basophils. Birds were given the SP had significantly reduced levels of circulating pro-inflammatory cytokines (IL-3, IL-6, IL-4, IL-18, and tumour necrosis factor), chemokines (CCL-20), and NOD-like receptor family pyrin domain containing 3 inflammasome than birds given the control diet.	(73)
*S. platensis* at 0, 100 g/kg	Feed/14^th^ -37^th^ day of age	Ross 708 male broiler chickens	LCP (17%) compared to basal diet (21%)/14^th^ -37^th^ day of age	The LCP-SP diet lessened the effects of the LCP diet, resulting in levels and ratios of monocytes that were similar to those of the control diet.	(74)
*S. platensis* at 0, 10%	Feed/15^th^ day-35^th^ day of age	Ross 708 female broiler chickens	Low protein diet (LCP) (17%)/15^th^ day-35^th^ day of age compared to basal diet (20%)/	Broilers fed SP in their diets saw considerably lower body weight gain but considerably higher feed conversion ratio compared to the other two treatments	(75)
Mycotoxins and other toxins					
*S. platensis* at 0, 0.02%	Feed/10^th^ day-45^th^ day of age	Commercial Broiler chickens	Aflatoxin (300 ppb)/10^th^ day-45^th^ day of age	SP fed to chicks who had been exposed to aflatoxin had positive benefits on their development, ready-to-cook yields, bursa weight, and cellular immune response, but SP had no impact on feed intake, feed conversion efficiency, leg abnormality scores, SRBC reaction, or the weights of the liver, giblets, spleen, or abdominal fat.	(72)
*S. platensis* at 0, 0.05%	Feed/8^th^ day – 42^nd^ day of age	Commercial Broiler chickens	Aflatoxin 0, 300 ppb B_1_/8^th^ -42^nd^ day of age	SP slightly mitigated the adverse effects of aflatoxin on body weight gain and the weights of the thymus and spleen, but not on the weights of the liver and kidney, proteins in the serum, or cholesterol.	(71)
*S. platensis* at 0 and 0.1%	Feed for three periods of 21 days each starting from 28th week.	Broiler breeder hens	Aflatoxin 0, 300, 400 and 500 ppb for three periods, each with duration of three weeks in broiler breeders from 28 to 36 weeks of age.	SP did not change the weights of the liver or the levels of kidney, proventriculus, or gizzard lesions in groups that were either fed alone or in conjunction with various amounts of AF.	(70)
*S. platensis* at 20 g/kg	Feed for 35 days	Cobb broiler chickens	-Deltamethrin/300 mg/kg diet for 35 days	Deltamethrin levels in meat, skin, and liver were all significantly lowered by SP, falling by 63.01, 63.00, and 62.90%, respectively. When compared to the group that got Deltamethrin, Sp increased protein and significantly lowered fat, cholesterol, and triglycerides.	(76)
*S. platensis* at 30, 60 and 120 mg/L	in drinking water daily for 90 days starting from day 15	Male ducklings	Arsenic trioxide:100 mg/L drinking water daily for 90 days	The not gained body weight in ducks was better in the arsenic and SP-treated groups (4.08-11.26%) than in the arsenic-only (14.93%). The drop in Total Erythrocyte Count, Hb, and PCV was less in the arsenic plus SP-treated groups than in the arsenic treated groups.	(77)
*S. platensis* at dose (10 gm/kg of diet)	in diet for two weeks after treatment (therapeutic) or before the treatment (preventive)	Broiler chicks	Hepatotoxic effect of diclofenac sodium: (2.5 mg/kg.b.wt., i.m)/at 21 days of age old for 3 days	In the preventative and treatment groups, the death rate dropped from 64% in the control birds to 8% and 32%, respectively. Preventive treatment improved lower haematological parameters, WBCs, absolute lymphocyte, eosinophil, and monocyte counts as well as decreased AST, ALP, uric acid, and cholesterol, as well as oxidative stress better than therapeutic group. Both SP-treated groups dramatically enhanced phagocytic activity, phagocytic index, and HI antibody against NDV	(44)
Heat stress					
*S. platensis* at 0, 0.5 and 1 g/kg diet	Feed from 4^th^ week – 14^th^ week of age	Gimmizah local Egyptian strain chicks (4 weeks of age)	Chronic heat stress condition (38°C ± 1; 55-65% RH)/3 days per week from 11.00 am to 15.00 pm/from 4^th^ week – 14^th^ week of age	Without affecting body weight gain or FCR, different Spirulina concentrations dramatically reduced the negative effects of heat stress on feed intake, the immune system, total lipids, LDL, WBCs, RBCs, albumin, globulin, creatinine, and liver enzymes (ALT, AST.	(78)
*S. platensis* at 0, 0.5 and 1%	Feed from 17^th^ day to 45^th^ day of age	Cobb 500 Broiler chickens	Heat stress (36°C for 6 h/d) from 38^th^ to 44^th^ day of age	Spirulina supplementation increased humoral immunity response and elevated antioxidant status while decreasing concentrations of stress hormone and several serum lipid markers. However, it had no appreciable impact on performance traits.	(79)
*S. platensis* at 0, 0.1, 0.3, and 0.5%	Feed for 6 weeks	laying Japanese quails (98 days old)	Heat stress (8h of 34 ± 1°C; 60-70% RH) for 6 days	Different Spirulina concentrations had no appreciable impact on feed intake, FCR, egg weight, and hen day egg production percentage. The lowest ileal E. coli count, blood MDA, heterophil, and H/L ratio was obtained significantly by SP at 0.5%.	(80)
*S. platensis* at 0, 0.5, 1 or 1.5%.	Feed from 21^st^ day to 42^nd^ day of age	Cobb-500 broiler chicks	Cyclic heat stress (34 ± 1°C for 8 h per day)	SP reduced the deleterious effects of heat stress on the final average daily increase, body weight, and FCR, with the chickens given 1% Spirulina showing the highest results. SP raised Hb, hematocrit and HDL levels and markedly reduced LDL and lipid peroxidation levels compared to stressed non-supplemented group. SP 0.5 or 1% enhanced carcass dressing, breast, and leg %	(81)
*S. platensis* at 5, 10, 15 and 20 g/L	Drinking water/in the morning (06:00–12:00 PM) for 6 weeks	White broilers	The experiment was conducted using a deep litter rearing system during the hot, humid summer months (for 6 weeks)	It had neither negative nor positive effects on the performance of broilers but had a considerable impact on Hb, RBCs, and shank pigmentation. SP by 15 and 20 g/L raised blood protein concentration and reduced serum fat content and transaminases. SP by 20 g/L significantly improved humoral immunity against ND vaccination and cell-mediated immunity to phytohemagglutinin-P.	(82)
*S. platensis* at levels of 0, 5, and 10 g kg-1 individually and in combination with selenium nanoparticles (SeNPs) at 0, 0.1, and 0.2 mg.kg-1	Feed for 5 weeks	Ross-308 broiler chicks	Heat stress (34 ± 2°C for 24 h for first 14 days. Then, 34 ± 2°C for 12 h (from 9:00 to 18:00) for three consecutive days a week and then 25 ± 2°C during the remaining experimental period).	Significant improvements were made in growth performance, blood lipid profile, carcass dressing, and carcass yield percentages thanks to SP and SeNPs combinations. Both supplements induced greater levels of the IgG, IgM, and IgA and rose antibody titers to IBD, AI, and ND quantitatively compared to the control group. All groups, except SP 5g, had elevated levels of glutathione peroxidase, superoxide dismutase, and blood triiodothyronine. The greatest favourable effects were obtained by SP 5g plus SeNPs 0.2 mg kg-1 and SP 10 g plus SeNPs 0.1 mg kg-1.	(83)

Dietary supplementation of *S. platensis* in broiler hens has shown to alleviate the adverse effects of high ambient temperature, including impaired enzymatic antioxidant system, raised stress hormone, and altered lipid profile (84). There was a dose-related modification of productivity, physiological, and immunological parameters when chickens under heat stress were given Spirulina in drinking water or feed (78, 82). Additionally, in ovo injection of *S. platensis* improved the broiler embryo’s ability to tolerate heat in the last days of incubation (22).

## Limitations of Spirulina as a feed additive and future directions for its use

Spirulina as a feed additive can have implications for productivity and end product quality, depending on the system of animal production. Both swine growth performance and product quality were not negatively affected in response to dietary supplementation with spirulina which might be attributed to lower protein requirements in finishing diets. However, Spirulina negatively affected chicken and fish production performance besides altering product quality, particularly meat color, according to the consumer’s opinions (85). One of major challenges regarding use of Spirulina as a feed addtitve at high level is that the gelation of its indigestible proteins causing birds to perform worse due to the increased digesta viscosity (32). Therefore the golden standard level of Spirulina inclusion into the feed should be highlighted and interpreted to be applied in the field. In addition, trials to increase digistability of Spirulina should be rescearced. Another major challenge regarding use of Spirulina as a feed addtitve is its quite higher cost compared to other protein ingredients such as soybean meal (58, 86). However, improving production efficiency and using waste streams as culture media, spirulina could replace fishmeal by becoming competitive for fish feed due to higher cost of fishmeal (86). Second major challenge with spirulina is limitation of its large scale production as presently it is produced at a smaller scale primarily for the nutritional supplement sector with few exceptions (58). Third challenge is with sustainable production of spirulina as compared to the other protein ingredients like soybean which is mainly attributed to the sensitivity of spirulina to the production system and regional climate (87). Therefore serious research and development efforts are required to improve yield of spriulina and make its production more sustainable. For example some research initiative targeted to improve sustainability of production by using biogas effluent (88) or wastewater (89) as production media. Additionally, waste heat sources (e.g. heat produced during biogas production) can be integrated as spirulina requires warm temperature (35–37°C) for cultivation (5, 90). Consequently, above mentioned challenges could be overcomed by upscaling and optimizing production of spirulina. Moreover, advanced techniques can also fascilitate improvement in yield and protein quality of spirulina through breeding and Finally, although spirulina has a high proportion of crude protein, improvements to protein quality could be possible through breeding and nutrition/production research (91). Therefore, future research focused on sustainable production and product processing and acceptance should investigate the trade-offs of incorporating spirulina into poultry diets.

## Conclusion

Bioactive metabolites are abundant in natural products and have been used for their medicinal properties. Spirulina that was regarded as a blue-green filamentous algae with a spiral shape, it has been identified as a genus of photosynthetic bacteria (Arthrospira) more recently. It is a highly nutritious and antioxidant natural product and having the ability to improve production performance either growth, hatchability, or egg production. The cell-mediated and humoral immune response as well as antimicrobial activities of spirulina promoted disease resistance and improved survival and growth rates in chicken. However, further studies on optimum dose of Spirulina for different poultry species, age groups, and production systems as well as the type of used Spirulina extract, organic or aqueous are required.

## Author contributions

All the authors contributed significantly to this manuscript. NAE conceptualized the manuscript. NAE wrote the first draft with input from SJ, MRF, MA, AAA, RA, KD, FH, and MAl. Authors NAE, SJ, MRF, MA, AAA, RA, KD, FH, and MAl. reviewed and updated the manuscript. All authors contributed to revisions and approved the final manuscript.
